# Cardiovascular and metabolic outcomes of GH replacement therapy in adults with GH deficiency – gender gaps

**DOI:** 10.1007/s11102-026-01646-0

**Published:** 2026-02-20

**Authors:** Angelo Milioto, Daniela Esposito, Gudmundur Johannsson, Oskar Ragnarsson

**Affiliations:** 1 Institute of Medicine, Sahlgrenska Academy, University of Gothenburg, Gothenburg, Sweden; 2 Department of Endocrinology, Sahlgrenska University Hospital, Gothenburg, Sweden; 3 Wallenberg Centre for Molecular and Translational Medicine, University of Gothenburg, Gothenburg, Sweden; 4 Department of Internal Medicine and Clinical Nutrition, Institute of Medicine, Sahlgrenska Academy, Department of Endocrinology at Sahlgrenska University Hospital, University of Gothenburg, Bruna Stråket 15, Gothenburg, SE-413 45 Sweden

**Keywords:** Growth hormone deficiency, Growth hormone replacement treatment, Sex, Hypopituitarism, Cardiovascular, Metabolism

## Abstract

Adult growth hormone (GH) deficiency is associated with increased body fat mass, abdominal obesity, dyslipidaemia, reduced exercise capacity, impaired cardiac function, reduced self-reported well-being, impaired quality of life, as well as increased morbidity. Randomised controlled trials and cohort studies, together with meta-analyses, have shown improved outcome in adult patients with hypopituitarism receiving GH, with a reassuring safety profile. Women with GH deficiency and hypopituitarism have greater morbidity and mortality than men, particularly in relation to metabolic and cardiovascular outcome. The response to GH replacement is also less favourable in women than in men. The reason for these differences in women and men with hypopituitarism is not entirely clear, but is likely related to the interaction between sex steroid and the somatotroph axis. In this narrative review we summarize the burden of GH deficiency in adults with hypopituitarism, and the impact of GH replacement, with focus on differences among women and men.

## Introduction

Growth hormone (GH) is a peptide produced by somatotroph cells of the anterior pituitary [1]. Growth hormone deficiency (GHD) in adults results from a damage of somatotroph cells, most commonly caused by a pituitary tumor, either due to mass effect or as a consequence of its treatment. GHD may also be caused by traumatic brain injury or, more rarely, by inflammatory or infiltrative disorders and infectious diseases affecting the pituitary gland [1–3].

**Fig. 1:**
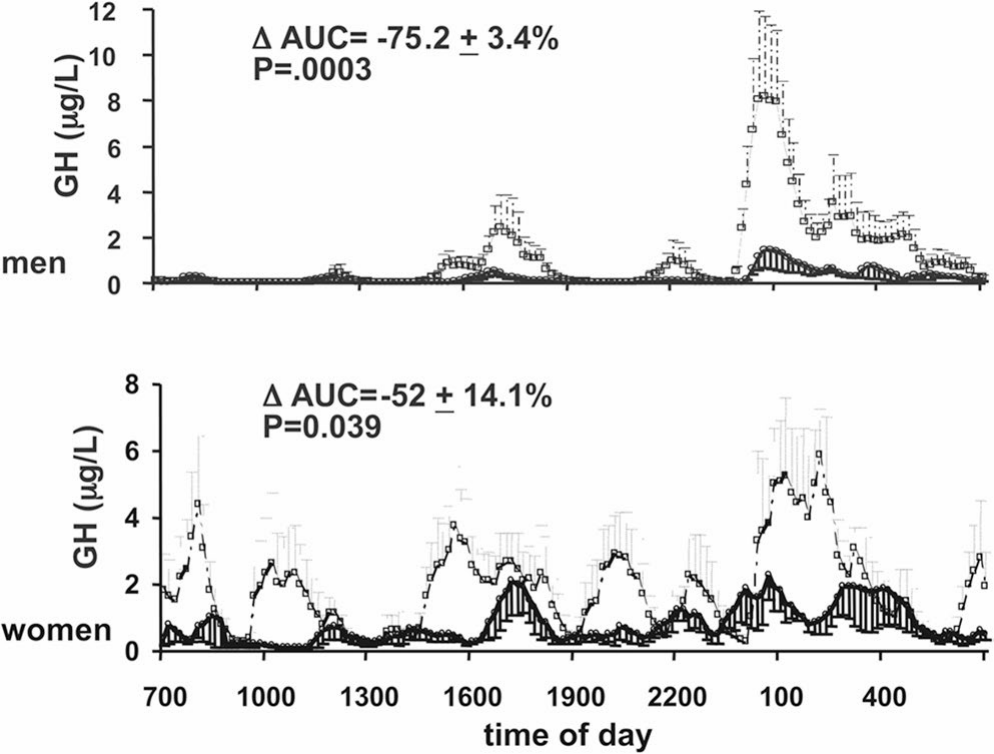
Twenty-four-hour growth hormone profiles in young adults. The graphs show mean ± standard error of serum GH concentrations over 24 h in six men (upper panel) and five premenopausal women (lower panel), aged 20–28 years, during saline infusion (open symbols) or GHRH antagonist infusion (closed symbols). Women exhibit larger and more frequent GH secretory bursts than men during both saline and GHRH antagonist infusion. In both sexes, GHRH antagonist infusion reduced the area under the curve compared with saline infusion (*p* = 0.0003 and *p* = 0.039 in men and women, respectively). Figure reproduced from Jessup et al. (https://doi.org/10.1210/jc.2003–030246 )

Under physiological conditions, the main role of GH in adults is to regulate metabolism, directing different substrates towards catabolic or anabolic processes [4]. Indeed, GHD leads to metabolic alterations such as abnormal body composition, an atherogenic lipid profile, insulin resistance and other metabolic complications that increase cardiovascular risk and contribute to the increased mortality [4].​ GH replacement therapy is the main treatment, as it improves the metabolic status of patients with GHD [5]. Notably, there are differences between men and women both in GH secretion under physiological conditions and in the response to GH replacement therapy (Fig. 1) [6].​

This review aims to summarise the effects of GH under physiological conditions, the consequences of GHD, and the impact of GH replacement therapy, with a specific focus on sex-related differences.

## GH secretion in women and men

The secretion of GH is pulsatile and takes mainly place in the fasting state, and during sleep at night [7]. GH secretion reaches a peak around puberty and declines with increasing age. It has been estimated that premenopausal women secret 1.5–3.1 times more GH than men, mainly due to higher GH-secretory burst amplitude [8, 9]. In premenopausal women spontaneous GH secretion is also higher during the periovulatory phase than the early follicular phase indicating a correlation between circulating estrogen concentrations and GH sercetions [8, 10]. The difference in GH secretion between women and men disappears after menopause. These data strongly suggest that estrogen augment endogenous GH secretion. Adult men and women, however, have similar concentration of serum IGF-I concentration [11] suggesting that estrogen inhibits the peripheral action of GH. Obesity, and in particular abdominal obesity, has a powerful negative impact on GH secretion [12]. GH secretion is also affected by nutrition and physical fitness [9, 10]. Thus, the mechanisms behind the reduced GH secretion seen with increasing age and the difference between men and women is complex and not completely known.

### Sex steroid regulation of GH action

In the adult, GH is a major regulator of substrate utilization, influencing fat and protein metabolism [13, 14]. GH stimulates lipolysis to enhance oxidative utilization of fat and is a potent stimulator of protein synthesis. GH therefore increases lean body mass and reduces fat mass. GH, through IGF-I, plays an important role in sodium homeostasis by stimulating renal tubular reabsorption of sodium that leads to an expansion of the extracellular water compartment. The anabolic actions of GH are mediated through IGF-I, whereas other metabolic actions such as lipolysis and induction of insulin resistance do not involve IGF-I. The GH-IGF-I axis is therefore a major regulator of body composition in humans.

There are major differences between men and women in their body composition with men having more lean tissue mass and less body fat mass than women [14]. Men have also more abdominal fat in relation to total fat than women. In women going though menopause both estrogene and GH concentrations decreases leading to reduced lean tissue mass and increased abdominal fat mass. This suggest an important interaction between GH secretion and sex steroids in the regulation of body composition.

Testosterone by it self stimulates lipolysis and attenuates lipogenes and therefore reduces fat mass as GH [15]. Testosterone has also proteinanabolic effects that act independently from the action of IGF-I. Oral estrogen, but not transdermal, reduces serum IGF-I concentration and increases GH secretion in healthy women [14]. Oral estrogen also reduces the hepatic fat oxidation and direct intrahepatic free fatty acids into a lipogenic pathway resulting in increased production and secretion of very low-density lipoprotein (VLDL) from the liver that is then available for peripheral lipogenesis. In line with these data, oral administration of estrogen causes a reduction in lean body mass and an increase in fat mass in comparison with transdermal administration.

These data suggest that oral estrogen has an inhibitory effect on the hepatic action of GH. There are two suggested mechanisms for this inhibitory action, estrogen reduces the hepatic expression of the GH receptor that is dose-dependent [16] and estrogen also inhibits JAK/STAT in the GH signalling pathway by increasing SOCS-2 which in turn inhibits the phosphorylation of JAK2 [17]. These may be some of the mechanisms that explain the different responses of GH replacement seen in women in comparisons with men.

## Diagnosing GHD in adults – impact of sex

A variety of conditions affecting the hypothalamus and/or the pituitary gland can lead to hypopituitarism and GHD. The most frequent causes are pituitary adenomas and their associated treatments [13]. In many pituitary disorders, GH is often the first hormone to be impaired, particularly in individuals with pituitary tumors or those who have received radiotherapy targeting the hypothalamic–pituitary region. Consequently, in patients with hypopituitarism where one or more anterior pituitary hormones are compromised, GH deficiency is likely to occur. GHD should therefore be considered in all individuals with hypothalamic–pituitary disorders or any form of hypopituitarism.

Because GH is released in pulses, a single random serum measurement cannot reliably be used. Instead, the GH secretory capacity of the pituitary gland is best evaluated by using a validated stimulation test (Table 1). Also, since there is a large overlap in serum IGF-I concentration between healthy individuals and GH deficient adults, IGF-I can only rarely be used to diagnos GHD. An exeption is, however, patents with multiple pituitary hormone deficiencies and low IGF-I concentrations. These patients can be considered to be GH deficient and a stimulation test is not necessary.

**Table 1:** Validated stimulation tests for the diagnosis of growth hormone deficiency in adults. Abbreviations: GHRH, growth hormone releasing hormone; BMI, body mass index

	Cut-offs	Contraindications	Notes
Insulin tolerance test	3 µg/L (≥ 40 years old) 6.1 µg/L (< 40 years old)	Ischemic heart disease, epilepsy, geriatric patients	Gold standard; hypoglycemia is unpleasant for the patient
GHRH + arginine test	11 µg/L for BMI < 25 kg/m^2^ 8 µg/L for BMI 25–30 kg/m^2^ 4 µg/L for BMI > 30 kg/m^2^	-	Patients treated with radiotherapy affecting the hypothalamus may show false normal results; GHRH is currently not widely available
Glucagon stimulation test	3 µg/L for BMI < 25 kg/m^2^ 1 µg/L for BMI > 25 kg/m^2^	-	Nausea, vomiting and delayed hypoglycemia can occur
Macimorelin test	2.8 µg/L	Patients receiving QT-prolonging drugs or CYP3A4 inducers	Accuracy similar to insulin tolerance test

The most reliable methods for confirming GHD are the insulin tolerance test, the arginine + GHRH test, the glucagon stimulation test, and the macimorelin test. These tests should only be used in an appropriate clinical setting, i.e. in individuals with established hypothalamic–pituitary disorder or in patients already with other anterior pituitary hormone deficits. The insulin tolerance test remains the reference standard for diagnosing GHD. Diagnostic GH cut-offs typically range from 3 to 6.1 µg/L, with higher thresholds applied to younger adults. Both the arginine + GHRH test and the glucagon test are influenced by BMI, as rising BMI reduces peak GH responses. Consequently, the arginine + GHRH test uses BMI-adjusted cut-offs, and for the glucagon test, a threshold of 1 µg/L has been advised in overweight or obese individuals, especially when the pre-test probability of true GHD is low, compared to 3 µg/L in normal-weight individuals [18–20].

The influence of sex on GH respons to provocation is limited. In one study, however, GH responses to GHRH + arginine stimulation and insulin tolerance test were examined in 39 and 27 healthy subjects, respectively. BMI showed a strong inverse effect on responses in both tests [21]. Women had greater GH peaks than men with GHRH + arginine, but this difference largely disappeared after adjusting for BMI. No sex differences were observed in the insulin tolerance test.

The oral GH secretagogue macimorelin has recently been authorized for use in evaluating adult GHD [22]. It is generally well tolerated, and its diagnostic performance closely matches that of the insulin tolerance test, offering a more convenient alternative for assessing GH status in adults. Age, BMI, and sex seem to have minimal impact on macimorelin’s diagnostic performance [23].

## Sex differences in cardiometabolic consequences of GHD and its treatment

GHD leads to multiple metabolic abnormalities, including adverse changes in body composition [5], disturbances in glucose and lipid metabolism [24], and an increased risk of non-alcoholic fatty liver disease [25]. In addition, GHD often coexists with other pituitary hormone deficiencies, which can contribute to the metabolic and cardiovascular burden observed in these patients. In women with GHD and co-existent secondary adrenal insufficiency and hypogonadism, a severe androgen deficiency may occur, which can further negatively impact insulin sensitivity, lipid profile, body composition and quality of life. The interaction between GHD and androgen deficiency may therefore represent an underappreciated contributor to cardiovascular risk and reduced QoL in women [26].

**Fig. 2:**
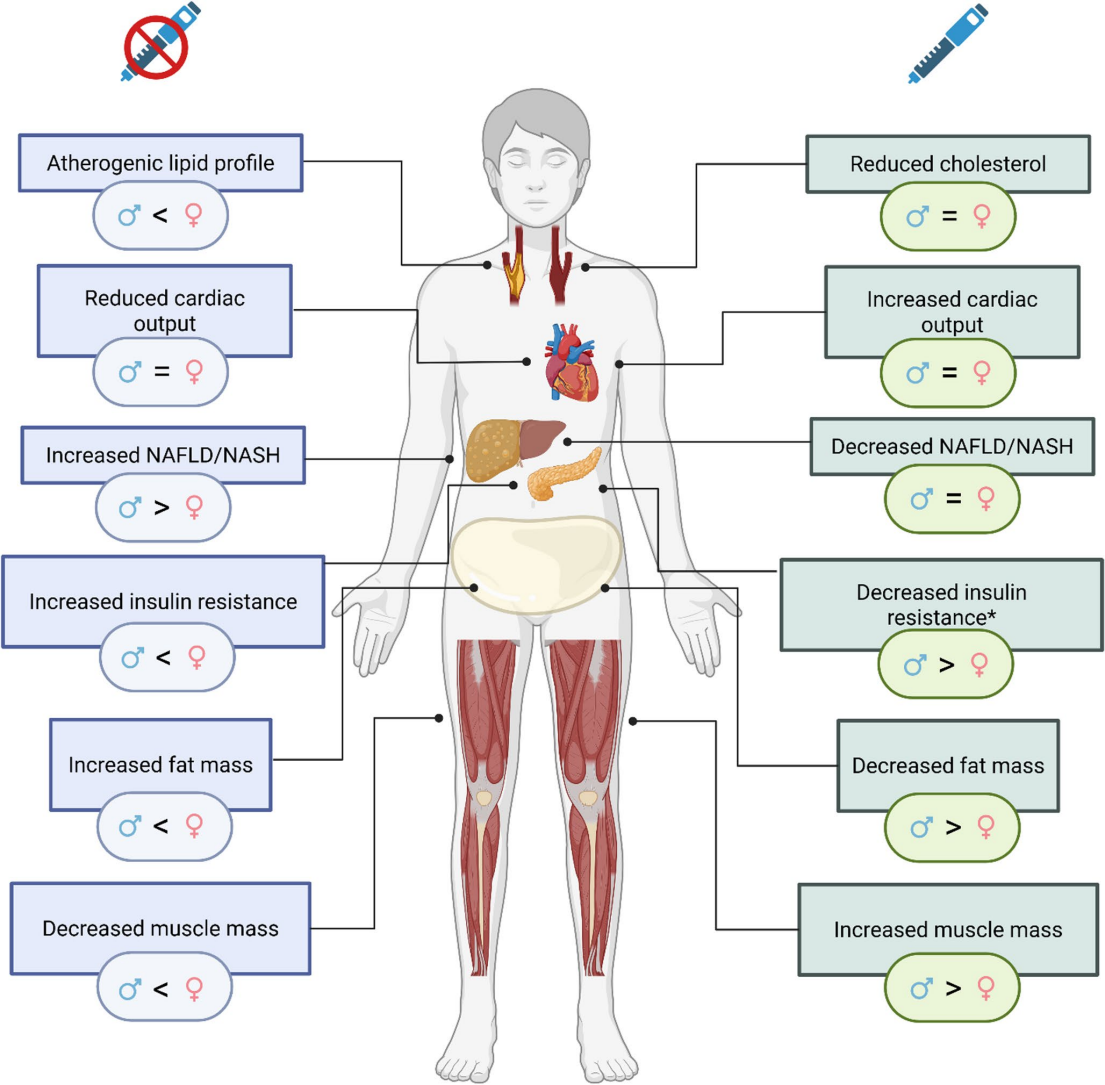
Cardiometabolic comorbidities of growth hormone deficiency in adults and the effect of growth hormone replacement treatment, with sex-related differences. *Evidence on the impact of GH replacement treatment on glucose metabolism are hetergenous. Greater-than and less-than symbols indicate higher frequency and/or severity of a given comorbidity in one sex compared with the other in the no-therapy panel (left) and a greater impact of the treatment in that sex in the therapy panel (right)

GH replacement therapy represents a cornerstone in the management of GHD, improving the metabolic profile through restoration of age-adjusted IGF-I [1]. Notably, differences between the sexes are observed before and during GH replacement therapy (Fig. 2). Women with GHD present with lower IGF-I concentrations at diagnosis than men [6, 27, 28] and, during GH replacement, the IGF-I increment at a given GH dose is smaller in women, indicating reduced GH sensitivity [6]. As a result, women generally require higher GH doses to reach comparable age-adjusted IGF-I than men [6, 29]. Oral estrogens, particularly ethinyl estradiol, further reduce hepatic sensitivity to GH and thus increase GH dose requirements, whereas transdermal estrogens will have a smaller impact on the IGF-I response [30, 31].

The GH dose requirement is higher among younger adults, higher in women than in men and the highest doses in order to achive normalisation of serum IGF-I are in women receiving oral estrogen replacement therapy. With increasing age the dose of GH needed to maintain IGF-I within the normal range reduces. After menopause the dose of GH in hypopituitary women not using estrogen replacement is similar as in men [32]. The treatment response in terms of changes in body fat and lean body mass follow the same pattern [33].

## Metabolic comorbidities

The most consistent clinical feature in GHD is the alteration in body composition, characterized by an increased fat mass, especially visceral fat, and a decreased lean mass [34]. In a study conducted on 2,589 patients, waist circumference at baseline was > 102 cm in 40% of men and > 88 cm in 56% of women [35]. In another study, bioimpedance analysis (BIA) showed that men and women with GHD had 6.1% and 5.3% higher body fat than controls, respectively [36]. When dual-energy X-ray absorptiometry (DEXA) was used instead, men with GHD had 4.4% and women 6.4% higher body fat compared to controls [36]. Patients with GHD demonstrated lower lean body mass compared to controls both when body composition was analyzed with DEXA and BIA [37–40]. Interestingly, GH replacement therapy was shown to decrease fat mass and increase lean mass, with men experiencing a greater reduction in fat mass [6, 41, 42] and a larger increase in lean mass [42, 43] compared to women.

Patients with GHD often exhibit dyslipidemia, with heterogeneous patterns across studies and between sexes, including elevated total cholesterol (TC), low-density lipoprotein (LDL), triglycerides (TG) and reduced high-density lipoprotein (HDL) [1]. In a cross-sectional study of 50 patients with GHD, men (*n* = 27) exhibited higher TC, LDL and TG compared to controls, whereas women (*n* = 23) had higher TG and lower HDL compared to controls [44]. In another study involving 30 patients with GHD (22 men, 8 women), both sexes showed lower HDL levels compared to controls, but only women had elevated TGL levels relative to controls [45]. GH replacement therapy improves the lipid profile in patients with GHD, although with sex-related differences. A long-term study (> 3 years) conducted on 2,981 patients with GHD, reported greater reductions in TC and LDL in women, more pronounced decreases in TG in men, and comparable increases in HDL levels in both sexes [46]. The beneficial effect of GH replacement on LDL levels can be explained by the physiological action of GH on the liver, where it upregulates hepatic LDL receptor expression and thereby increase the LDL clearance from the circulation [47]. GH replacement treatment may also increase HDL by stimulating hepatic production of VLDL, which in turn enhances VLDL–LDL turnover [48]. Moreover, by reducing the activity of lecithin: cholesterol acyltransferase and cholesteryl ester transfer protein, GH may decrease cholesterol esterification therefore contributing to increased HDL levels [49]. In summary, men with GHD have higher TC, LDL and TG levels, whereas women with GHD more often show reduced HDL and elevated TG. During GH replacement, women show greater reductions in TC and LDL while men have a more pronounced decreases in triglycerides, with comparable increases in HDL in both sexes.

Impaired lipid metabolism in patients with GHD is associated with an increased prevalence of non-alcoholic fatty liver disease (NAFLD) and non-alcoholic steatohepatitis (NASH) [50]. A recent meta-analysis reported that the prevalence of NAFLD in men with GHD was 60% and 51% in women, while the prevalence of NASH was 24% in men and 19% in women, suggesting a greater hepatic involvement in men than in women with GHD [51]. GH replacement therapy has been shown to exert beneficial hepatic effects, leading to a significant reduction in liver enzyme levels in patients with GHD and NASH treated for two years [52]. Moreover, GH replacement therapy normalized liver enzymes in 65% of patients (20/31) who had elevated transaminases at diagnosis and sex did not emerge as a predictor of treatment response [52].

Beyond lipid metabolism, glucose metabolism is also frequently impaired in patients with GHD. A large observational study of 6,050 adults with GHD reported a prevalence of diabetes mellitus of 9.3% compared with an expected prevalence of 8.2%, corresponding to a standardized prevalence ratio (SPR) of 1.13 (95% CI, 1.04–1.23). When stratified by sex, observed versus expected prevalence was 8.9% vs. 8.6% in men and 9.7% vs. 7.9% in women; SPR was increased only in women (1.23; 95% CI, 1.09–1.38) and not in men (1.04; 95% CI, 0.92–1.17), with a statistically significant difference between the SPRs in the two sexes [53]. Studies investigating the effects of GH replacement therapy on glucose metabolism show heterogeneous results. A study conducted on patients with GHD under GH replacement therapy for a maximum of 18 years, showed a statistically but not clinically significant increase in fasting glucose [54]. Similarly, a nationwide Swedish study of patients on GH replacement therapy for 15 years reported a significant increase in fasting glucose (from 4.4 to 4.8 mmol/L), whereas HbA1c decreased over the same period (from 31 mmol/L to 27 mmol/L) [5]. Interestingly, a study conducted in both the United States and Europe on patients with GHD treated with GH replacement therapy found that the incidence of diabetes mellitus was higher than in the general population in the United States, whereas in France, Germany and Sweden it was similar to that of the respective reference populations [55]. Finally, in a cohort of patients with GHD treated with GH replacement therapy, female sex, higher body mass index, younger age, a longer interval between pituitary diagnosis and GH replacement therapy initiation and shorter treatment duration emerged as significant risk factors for incident diabetes [56].

## Cardiovascular comorbidities

GHD increases the risk of cardiovascular diseases, particularly in women [57], due not only to the higher burden of metabolic comorbidities but also to the direct effect of GHD can on cardiac and vascular function. In fact, the endothelial production of nitric oxide, a vasodilator, is significantly impaired in GHD and this dysfunction contributes to increased peripheral vascular resistance and greater arterial stiffness [58]. Those mechanisms may partly account for the increased prevalence of hypertension observed in this population. In a large Spanish cohort of untreated patients with GHD, hypertension was diagnosed in 22.6% of men and 21.7% of women (overall 22.1%), compared with 14.9% in the reference population [59]. Interestingly, among patients with GHD not receiving antihypertensive treatment, men exhibited higher systolic blood pressure, whereas diastolic blood pressure was comparable between sexes [46]. Consequently, the increased prevalence of hypertension, together with the unfavorable metabolic profile, contributes to the higher prevalence of atherosclerosis observed in patients with GHD [60]. Additionally, the vascular alterations contribute also to structural and functional cardiovascular abnormalities, representing another clinical manifestation of GHD. In fact, studies on functional parameters demonstrate impaired myocardial performance with reduced left ventricle mass index, a decreased stroke volume and a diastolic dysfunction with a lower end-diastolic volume index [61–63]. Also, women have more pronounced diastolic dysfunction compared to men [24]. GH replacement therapy is associated with improvements in cardiac structure and function, as demonstrated across multiple studies, showing increase of left ventricular mass index [64–67], improved left ventricular [68, 69] and diastolic function [67, 70]. No sex-related differences were observed in the increase of left ventricular mass index and, in general, in enhancement of systolic function; however, men showed a greater improvement in diastolic function compared with women [24]. Nevertheless, during GH replacement therapy, the incidence of non-fatal cardiovascular and cerebrovascular events is comparable between sexes [46].

### Quality of life

Patients with GHD have impaired quality of life (QoL), with evidence demonstrating sex-related differences both in untreated patients and in those receiving GH replacement therapy.

Large-scale data have revealed that untreated women with GHD have worse QoL compared to men, that was evident across multiple patient subgroups, including those above and below 65 years of age, patients from different countries and those with various etiologies of GHD [71]. Concerning treatment, women with GHD show greater absolute improvements in QoL after 12 months of GH replacement therapy compared to men, however this greater beneficial effect observed in women reflects their worse baseline status, as both sexes demonstrate similar relative improvement during treatment [72]. Furthermore, an observational study reported a higher prevalence of sexual dysfunction in women with GHD compared with men. However, sexual dysfunction questionnaire scores were associated with poor QoL in men, but not in women [73]. Larger QoL improvements occur within the first year of therapy, with continued and gradual enhancement over subsequent years in both sexes [74].

## Mortality

The somatotropic axis is often the first and most frequent axis to be affected following damage of the pituitary gland. For this reason, in the past, the terms “hypopituitarism” and “growth hormone deficiency” were frequently used interchangeably, although it is now well recognized that deficiency of each pituitary axis displays distinct clinical characteristics [75]. Nevertheless, some studies on hypopituitarism have not included a thorough evaluation or definitive diagnosis of GHD.

A study published in 2001 involving 1,014 patients with hypopituitarism demonstrated an excess mortality among those who were not treated for GHD compared with the general population with a standardized mortality ratio (SMR) of 1.87, mainly due to cardiovascular and cerebrovascular causes. Notably, the excess mortality was higher in women than in men (SMR 2.10 and 1.42, respectively). However, only 112 patients in that cohort had been tested for GHD, and among the 98 diagnosed cases, 24 received GH replacement therapy [76]. Two more recent studies examined mortality among patients with a well-defined diagnosis of GHD who were not treated with GH replacement therapy [77, 78]. Neither study found an excess mortality compared with the general population. However, one study was limited by a small sample size (109 patients) [77] and the other by a short follow-up period (2.3 years) [78], both of which may have reduced the statistical power to detect differences in mortality.

A meta-analysis published in 2015 pooled mortality data from over 19,000 patients with hypopituitarism, regardless of whether GHD was diagnosed or not [79]. Across all studies, hypopituitarism was associated with increased mortality, with a pooled SMR of approximately 2.0 compared with the general population and women exhibiting higher mortality than men (SMR 2.53 vs. 1.71). When stratified by treatment, cohorts receiving GH replacement therapy showed lower mortality (SMR 1.15 vs. 2.40), approaching a mortality risk similar to the general population. However, men on GH replacement had SMR similar to general population whereas women on GH replacement continued to exhibit increased mortality of approximately 50% [79].

In a population-based cohort of adults with non-functioning pituitary adenomas, 207 individuals received GH replacement therapy, while 219 did not [80]. Patients receiving GH replacement therapy demonstrated lower mortality than the background population (SMR 0.65), whereas untreated patients had an SMR of 1.16. When analyzed by sex, men on GH replacement therapy showed a significant reduction in mortality (SMR 0.63). GH replaced women also had SMRs below 1, although not statistically significant. In contrast, untreated men and women exhibited slightly elevated SMRs, but these increases did not reach statistical significance [80].

In summary, current evidence indicates that patients with GHD have increased mortality, which can be mitigated by GH replacement therapy. The benefit appears greater in men than in women, possibly reflecting suboptimal GH dosing in women, who generally require higher doses to achieve comparable IGF-I levels.

## Conclusions

Growing evidence highlights important sex-specific differences in adults with GHD. Women with GHD display a higher risk of metabolic and cardiovascular complications and greater impairment in quality of life and sexual function compared with men. The underlying mechanisms responsible for these sex differences remain poorly understood. Potential explanations include biological differences in GH/IGF-I axis regulation, androgen deficiency, and sex-related disparities in diagnosis and management. Undertreatment with GH replacement therapy may also occur, as women with GHD usually require higher GH doses to achieve comparable IGF-I levels and overall response. Awareness of these differences should prompt clinicians to adopt a more individualized, sex-informed approach to diagnosis, hormone replacement strategies, and long-term monitoring in patients with GHD.

## Data Availability

No datasets were generated or analysed during the current study.
